# The Current State of Optical Sensors in Medical Wearables

**DOI:** 10.3390/bios12040217

**Published:** 2022-04-06

**Authors:** Erik Vavrinsky, Niloofar Ebrahimzadeh Esfahani, Michal Hausner, Anton Kuzma, Vratislav Rezo, Martin Donoval, Helena Kosnacova

**Affiliations:** 1 Institute of Electronics and Photonics, Faculty of Electrical Engineering and Information Technology, Slovak University of Technology, Ilkovicova 3, 81219 Bratislava, Slovakia; niloofar.esfahani@stuba.sk (N.E.E.); michal.hausner@stuba.sk (M.H.); anton.kuzma@stuba.sk (A.K.); vratislav.rezo@stuba.sk (V.R.); martin.donoval@stuba.sk (M.D.); 2 Institute of Medical Physics, Biophysics, Informatics and Telemedicine, Faculty of Medicine, Comenius University, Sasinkova 2, 81272 Bratislava, Slovakia; 3 Department of Simulation and Virtual Medical Education, Faculty of Medicine, Comenius University, Sasinkova 4, 81272 Bratislava, Slovakia; 4 Department of Genetics, Cancer Research Institute, Biomedical Research Center, Slovak Academy Sciences, Dubravska Cesta 9, 84505 Bratislava, Slovakia

**Keywords:** optical sensors, wearable, physiology, photoplethysmography, optical fiber, colorimetry

## Abstract

Optical sensors play an increasingly important role in the development of medical diagnostic devices. They can be very widely used to measure the physiology of the human body. Optical methods include PPG, radiation, biochemical, and optical fiber sensors. Optical sensors offer excellent metrological properties, immunity to electromagnetic interference, electrical safety, simple miniaturization, the ability to capture volumes of nanometers, and non-invasive examination. In addition, they are cheap and resistant to water and corrosion. The use of optical sensors can bring better methods of continuous diagnostics in the comfort of the home and the development of telemedicine in the 21st century. This article offers a large overview of optical wearable methods and their modern use with an insight into the future years of technology in this field.

## 1. Introduction

The development of mankind reflects the efforts of doctors, scientists, and others to maintain and strengthen health, to implement social measures and prevent disease. New and improving diagnostic methods for real-time and long-term health monitoring are constantly being established. Early accurate diagnosis is the key to maintaining a high quality of life [1,2]. Older methodologies based on invasive sampling with the use of heavy equipment are nowadays being transformed into simple scanning methods that do not require demanding manipulation and also make people feel more comfortable [3]. With the advancement of technology, miniaturization, the development of advanced materials, and the advent of the internet, wearable electronics are gaining prominence [2]. As the healthcare regime moves more toward personalized medicine, the wearable medical market is projected to grow by around 26.4% worldwide to $195.57 milliards between 2020 and 2027 [4]. The arrival of intelligent and wirelessly connected wearable monitoring devices brings a revolution in healthcare. The trend began with simple fitness straps and developed rapidly in the form of variable advanced health accessories such as watches, smart clothing, glasses, contact lenses, rings, and various body extensions and inserts [5,6]. These devices can closely monitor life functions, human health, and report long-term a change in the patient’s health indicators. Ideal wearable sensors must be non-invasive, compact, easily portable, easy to manufacture, and low cost [7,8,9]. However, human health monitoring with wearable electronics has its pitfalls, and the sensory principles often differ significantly from conventional laboratory measurements. There are hundreds of these sensory principles and they have been described in many publications [8,9,10], but on many occasions they do not consider their use in real life. In our article, we decided to focus more deeply only on a narrow group of promising sensors, specifically those that use optical phenomena for detection. We deal with basic physiological parameters and quantities measurable using optical wearable electronics, and their relationship to human health. In the following chapters, we describe the types of optical sensors and the latest selected and most promising trends.

### 1.1. Advantages of Optical Measurement

**Figure 1:**
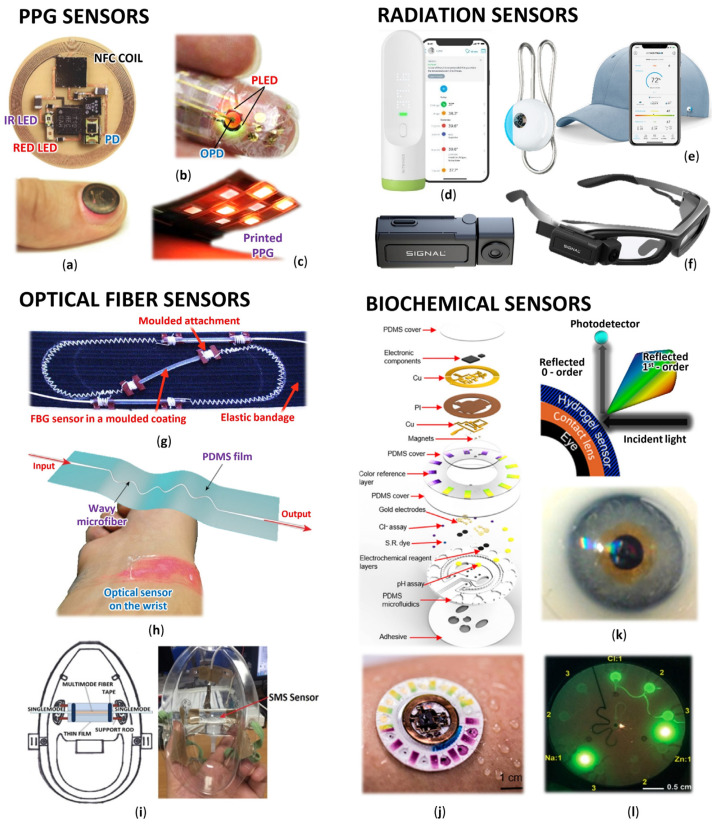
Wearable optical sensors for measuring of human physiology: (**a**) Miniaturized battery-free NFC enabled wireless systems for wearable pulse oximetry (unencapsulated device and device during operation mounted on a thumbnail) [12]; (**b**) Ultra-flexible organic PPG sensor attached to finger (smart e-skin system) [13]; (**c**) Printed reflectance oximeter array composed of four red and IR OLEDs and eight OPDs placed on the forearm for 2D oxygenation mapping [14]; (**d**) Contactless infrared medical grade thermometer composed from 16 IR sensors for forehead measurement with smartphone connectivity [15]; (**e**) Battery-free skin UV exposure tracker in form of fashion clip button with smartphone connectivity [16]; (**f**) Smart glasses with thermal camera for precise temperature measurement and scanning ideal for medical, industrial, and environmental use [17]; (**g**) Respiration belt with embedded silica fiber optical sensor for thoracic movement analysis [18]; (**h**) Self-assembled wavy optical microfiber for stretchable wearable sensor (schematic diagram and sensor stuck on the wrist) for monitoring of radial artery pulse wave [19]; (**i**) Optical fiber interferometer based breathing sensor built into oxygen mask [20]; (**j**) Skin-interfaced microfluidic battery-free systems for simultaneous electrochemical, colorimetric, and volumetric analysis of sweat [21]; (**k**) Contact lens integrated glucose monitoring using smartphones [22]; (**l**) Skin-wearable fluorometric microfluidic device (emitted by blue light) for measuring of Cl, Na and Zn sweat concentrations [23].

Optical sensors are, in principle, detectors that capture the physical amount of light or its variations. In our article, we focus on optical sensors that enable continuous and highly sensitive measurement of parameters about our health and the environment for medical diagnostics and physiological health assessment [11]. Such progressive sensors are manufactured applying fundamental optical technologies such as photoplethysmography (PPG), optical fibers with Bragg gratings (FBG), interferometers often woven in smart textiles, various radiation sensors, plasmonic and fluorometric sensors, and colorimetry, as well as the development of prospective new materials and organic components (Figure 1).

Optical sensors are currently gaining great recognition and are becoming an increasing alternative to traditional electrical or mechanical sensors. Overall, they offer unique advantages in recording human health compared to electrical sensors. They propose excellent metrological properties such as low zero and low sensitivity drift, good accuracy, sensitivity, and large usable bandwidth [24]. They are immune to electromagnetic interference, electrically safe, can achieve outstanding miniaturization, and they are capable of capturing nanoscale volumes, allowing the non-invasive examination of biological matter with relatively large penetration depths. Sensing elements, whether in the form of optical fibers or optically transparent encapsulated photoplethysmographic and biochemical sensors, are often inexpensive, water- and corrosion-resistant [25]. As microelectronic technologies are driven by requirements for wearable devices towards higher sensitivity, faster response, better robustness, and higher integration, they may ultimately reach their limits, which are inherent in the very nature of low-frequency electromagnetic fields [26,27,28,29,30,31]. The response time is limited by parasitic effects and in high-density electronic circuits by signal crosstalk. These limitations can be avoided by using photons as a signal carrier [32]. In many cases, the optical sensors do not even have to be in direct contact with the human body or do not require a high quality of contact. Other significant advantages include the possibility of implementing distributed sensors, which allow quantities not only to be read but also to be transmitted directly. Due to these features, optical sensors become a great and advanced solution for monitoring physiological parameters with wearable devices and for medicine in general. Today, about 15% of the market for wearable devices is based on optical sensors and this number is constantly growing [24]. Another not negligible benefit is the great progress of flexible technologies in the field of optical sensors, thus the newly discovered highly flexible and soft optical sensors are expected to provide a reliable and safe alternative for the next generation of intelligent wearable medical devices. Organic semiconductor devices have many attractive properties. These include simple production on flexible substrates, the possibility of miniaturization, the simultaneous ability to generate and detect an optical signal, and tunable light emission in a wide range of values [33,34,35]. The potential of optical sensors in medicine has even begun to be explored. They serve in this field not only as sensors but also as manipulators of biological activity, for example, biosensors such as lab-on-chip spectrometers [36,37], plasmonic spectrometers [38], and flexible e-skin [39,40,41].

### 1.2. Health and Optical Wearables

Optical sensors in medicine have a broad spectrum of capabilities. They can be used to increase the intelligence of medical equipment, implants, and to monitor human physiology remotely even without direct contact with the patient. Monitoring the processes in the human body allows easier detection of vital signs such as heart rate (HR), respiratory rate (RR), blood pressure, etc., allowing early rapid diagnosis and prevention to be performed, and generally helping people to monitor their physiological parameters and inform the doctor in case of change.

HR measurement is a common method for determining the physical activity and condition of the body. It can predict cardiovascular morbidity and mortality in a very reliable and easily accessible way. HR reflects the overall activity of the autonomic nervous system and provides a suitable indicator of a person’s condition and mood. HR variability (HRV) is derived from HR and is a time variance between heartbeats. It is a good sign of physical fitness. When the HRV is high, the nervous system is balanced, and the body can adapt to the environment and function well. Low HRV indicates that the body is working hard, it is tired, dehydrated, stressed, or sick [42,43]. As the HR arises as a wave in the blood vessel walls caused by tensioning and accelerating blood flow, it then spreads from there through other arteries throughout the body. These changes can be easily and reliably detected by optical sensors, mostly PPG or optical fiber.

Respiration monitoring is also a crucial physiologic parameter in inpatient examination. Breathing, along with pulse, blood pressure, and body temperature, is one of the vital signs. Healthy, normal breathing is regular, evenly deep, soundless, and odorless. The respiratory impulse increases with decreasing oxygen content, increasing carbon dioxide content, and decreasing pH. Deviations may indicate certain diseases, such as anxiety and potential hypoxia. Respiratory disorders occur not only in respiratory diseases but also in cardiovascular diseases and metabolic disorders. RR monitoring is important in detecting symptoms of sleep apnea, chronic obstructive pulmonary disease, asthma, or children’s pulmonary diseases [44]. RR can be measured using various devices and physiological principles such as spirometry, capnometry, impedance pneumography, acceleration sensors, etc. Today, a very progressive method is the algorithms’ extraction from the captured photoplethysmography signal.

Blood pressure (BP) demonstrates the pressure exerted by the blood on the arterial wall, which provides information on blood flow during heart contraction (systole) and relaxation (diastole), and may also indicate cellular oxygen supply. Its value is affected by cardiac output, blood viscosity, vascular elasticity, and resistance. Hypertension is a modern epidemic and is the most important risk factor for cardiovascular disease, leading to an increase in overall mortality. BP is traditionally measured using inflatable pressure cuffs, but this is completely impractical in wearable electronics. Thus, great efforts are made to measure BP based on the pulse wave transition time (PTT), whether from the shape of the PPG curve or the time shift between ECG and PPG in the periphery [44].

Body temperature (BT) is essential for maintaining all vital functions and metabolic processes. In humans, BT is usually constant, but various external and internal influences, especially inflammation, can affect it. From a medical point of view, the measurement of BT is very important, because many diseases are accompanied by characteristic changes. Different values of the temperature control center are related to the immune response of the organism defending itself against the progression of the disease. BT is divided into body core and peripheral temperature, where the peripheral temperature is more variable. The temperature is affected by blood circulation, HR, stress, metabolism, and external microclimatic factors [44]. Among the innovative methods of measuring BT is the use of radiation sensors or optical fibers.

Human fluids also offer an important source of information about the human body. Non-invasive and continuous measurement of biomarkers such as sodium, chlorine, potassium, lactate, calcium, glucose, ammonia, ethanol, urea, cortisol, and various neuropeptides and cytokinesis is possible from sweat or saliva. For example, excessive drinking leads to hyponatremia (low serum sodium) and conversely, hypohydration leads to a higher risk of disease (e.g., dementia) and body failure (e.g., sunburn). Proper hydration is important and therefore measurements and warnings of non-compliance can help prevent the associated problems [45]. Lack of drinking can be easily detected from urine or blood, but collection cannot be performed while moving, but this measurement problem can be solved with the help of optical sensors together with a set of sensors that allow to measure the indirect method of hydration [46]. Various biochemical optical sensors are most useful in this area.

The incidence of diabetes mellitus is growing to epidemic proportions in today’s developed world. Continuous monitoring of blood glucose levels is important for people with this disease. In this case, the sensors offer a non-invasive method of continuous measurement, unlike current glucometers, which require blood collection from a finger. The accuracy of home glucometers also varies ±15%. The development of optic sensors today focuses on the rapid, continuous and simple determination of glucose in surrounding body fluids, for example, the use of a photonic crystal that responds to glucose levels has been described, the sensor bends light, and the diffracted band shifts as the glucose concentration changes [47].

In today’s modern age, the total physical activity of the population is falling below the recommended levels, so the incidence of population-related diseases such as obesity and diabetes is increasing. Monitoring of childhood obesity is known, where accelerometers offer objective measurement of normal activity independently of self-report [48,49], or the monitoring of seniors in in-home care, where the system records their activities, events, and potentially important health symptoms [50,51]. Nowadays, sensors based on optical sensors are coming to the forefront that help to better monitor patients, which helps to improve the quality of life.

## 2. Photoplethysmography

In healthcare, one of the most widely used optical imaging methods is photoplethysmography (PPG) (Table 1), which is used to monitor blood flow in real-time. It is used to determine the physiological parameters such as oxygen saturation, blood pressure, cardiac output, respiration but also to evaluate autonomic function, depth of anesthesia, as well as detection of peripheral vascular disease [52]. In essence, this method can measure changes in volume throughout the body. It measures the amount of light that is absorbed or reflected by blood vessels in living tissue. As blood flows, a cardiovascular pulse wave emanates from the heart, propagating through the body and periodically dilating the arteries and arterioles in the subcutaneous tissue. The PPG signal is a mixture of blood flow in the veins and arteries and generally consists of a pulsating and non-pulsating component of the blood volume [53]. The pulsating component is related to changes in arterial blood volume and is synchronous with the heartbeat, while the non-pulsating component is a function of basal blood volume, respiration, sympathetic nervous system activity, and thermoregulation [54]. Light in the spectrum from the visible to the near-infrared (NIR) region can only reach human tissues to a depth of a few millimeters, and its penetration is limited due to the absorption of light by blood, melanin, fat, water, and light scattering. The actual interaction of light with the tissue depends not only on the composition of the tissue but also on the wavelength. These differences allow the detection of different information from the PPG signal. For example, green light is immediately absorbed by the body, so it is only suitable for measuring in places where a lot of blood is in the tissue, but it is less affected by ambient light interference. On the contrary, red light and NIR penetrate deeper into the human body and thus provide a wider range of information about the physiological signal [55,56]. This phenomenon is also related to the composition of the skin and evolutionary development, where the epidermis performs a protective function of the underlying soft tissue against harmful UV radiation. Skin pigmentation absorbs shorter wavelengths, UV, and to some degree also visible light. Water in tissues, on the contrary, absorbs in areas with long wavelengths. In the literature is also mentioned an “optical window” for wavelengths of 600 to 1300 nm [57,58,59].

The PPG signal can be measured in two geometric configurations. If the light-emitting diodes (LEDs) and the photodiode (PD) are facing each other and the light passes through the tissue, where we measure the non-absorbed light, we speak of the transmission principle. This configuration is most suitable for areas with high capillary density, such as fingers or the earlobe. Red (680 nm) or near-infrared (810 nm) lights are usually used and deep penetration is required [60,61]. Such measurements are more stable, repetitive, and less sensitive to position changes. Transmission PPG configurations increase the perfusion index by 40 dB to 60 dB [45,62]. If the LED and PD are on the same side and use reflection from the internal structures of the skin, we speak of reflective PPG [63,64,65,66,67]. This configuration has a lower signal quality, but it is more suitable for nonstop wearing. Since the maxima of the pulsating component of the reflected light occurs in the range between 510 and 590 nm [68], the green (565 nm) or yellow (590 nm) light is usually used [69]. 

The PPG measurement can be performed in all parts of the human body, which provides sufficient availability of blood vessels and a sufficient pulsating component, especially when using the reflective method. Very common used areas for the reflective method are around the wrist [3,70], mostly in the form of various smart bracelets and watches, at the forehead [71], areas around the biceps, and the calf muscles or chest [72], often in the form of patches. In medicine, the fingers and ear lobes are very often used, where devices in the form of pliers working on the transmission principle are used. When designing the sensor, we must consider that the skin structure may slightly differ depending on the person and location on the body. Age, gender, and pathological conditions can cause various reflections, scattering, and light absorption [73]. For example, if you try to use a PPG sensor optimized for measurement on the wrist (in a smartwatch) and place it on the chest, you will find that the optical performance is insufficient and the sensor does not reach the required penetration. Some progressive PPG sensors are introduced to the use of organic materials. There is a systematic study of the reflective PPG sensor, where different printed OLEDs (RED and NIR) were utilized and compared, and the organic photodetector geometries, spacing, and barriers to maximize sensor performance. Finally, they also used inverse-variance weighting and template matching algorithms to improve the HR detection from the multichannel PPG signals [74]. A comprehensive review of the most up-to-date wearable PPGs was described by Daniel Ray et al. [45], who focused on multi-wavelength PPG sensing, technology, physiological parameter estimation, motion artifact reduction [75], and recommendations for standardization and overall theoretical details. Tarara et al. [73] described their challenges, and Bent et al. [76] examined the sources of inaccuracy such as different skin types, movement, and signal crossover. PPG technology is very successful but still not ideal. There are still challenges and improvements to be made. PPG is not fully reliable, the limitations are in the measurement’s standardization, the setting up of regularizing ranges of data to correlate with patients, and the evaluation of different treatment actions.

### 2.1. Heart Pulse Measurement

The skin consists of seven main layers, and each layer exhibits a different thickness, absorption, and scattering coefficients. The layer that determines pulse detection is the sixth layer called the inferior blood net dermis layer. For reliable detection in these skin layers, the used LEDs (respectively, light sources) and photodiodes PDs must ideally operate in the wavelength range of the absorption peaks of blood hemoglobin and deoxyhemoglobin, i.e., between 540 and 570 nm [77]. PDs and the LEDs must be optically isolated [78], for example, using a raised mesa, and the whole optical system must be surrounded by optical separation techniques to reduce the impact of ambient light [79]. For a more reliable result, modern chips also include an ambient light sensor. The PPG signal, when recorded in a real environment, often contains movement artifacts and is also affected by the changing pressure of the sensor on the skin [80]. To minimize these artifacts, accelerometric sensors or multiple optical paths using two photodiodes are therefore included in modern PPG chips. The output of the accelerometer and the two PPG signals are then processed using motion compensation algorithms, a Kalman filter, and an adaptive notch filter [81]. HR measuring using PPG is well characterized and works seamlessly; however, we must take into account that the amount of data processing for healthcare applications is large and requires considerable computing power and continuous energy supply [11].

Ishikawa et al. [82] introduced the PPG HR sensor, which overcame motion artifacts, in the form of a wristband that can be worn daily during various activities. A FIR filter was used to cancel arm-related motion artefact, and for finger and wrist-related motion a band-pass filter based on the body tissue HR was used. Finally, pulse noise-free detection signals were obtained using peak detection and autocorrelation methods. This calibration achieved noise-free HR detection [78]. Tison’s group has made progress in the diagnosis of atrial fibrillation (AF). They used a PPG sensor and an accelerometer of a commercial smartwatch to obtain HR data and the number of steps. Thanks to the algorithm based on heuristic pretraining and deep neural network training, their device can detect the fibrillation with sensitivity of 98% and specificity of 90.2% [83]. An interesting project was the smart pulse sensing glasses with Bluetooth low energy connectivity designed by Nicholas Constant et al. This device used a PPG sensor located on the nose for unobtrusive and continuous monitoring of HR [84]. Devices using NFC communication represent a very promising version of HR monitors [85]. In this particular case, it can also measure temperature via a thermistor. The sensing device is activated only when a reader-device, such as a smartphone or tablet, is approaching, for example during a call. The reader-devices supply all the necessary energy for measurement via a coil at the edge of the sensing-device. It resembles contactless ATM cards, only instead of sending data about your identity and bank transaction, it performs a quick physiology measurement [86]. Such devices represent an interesting alternative to conventional battery powered devices. Optical sensors for HR detection can be in the form of wide variety of daily wear items that do not interfere with humans and fulfill their diagnostic role. 

### 2.2. Detection of Blood Oxygen and Glucose

Arterial oxygen saturation (SpO_2_) indicates the percentage of the total hemoglobin in the blood that is saturated with oxygen. The SpO_2_ provides information on lung efficiency and is an important indicator of overall human health. These measurements are similar to those using LEDs and PDs, but the estimation of SpO_2_ level is based on the different spectral characteristics of hemoglobin and deoxyhemoglobin, thus two LEDs are used. Deoxyhemoglobin absorbs red light better than oxyhemoglobin, while oxyhemoglobin absorbs infrared (IR) radiation better. Blood oxygenation is proportional to reflected red light (622–780 nm) and IR (780–2400 nm) [87]. However, some researchers have identified that orange and green lights perform better because of their resistance to motion artifacts [88,89]. A very promising design is the flexible concept (Figure 1a) [12], which incorporates advanced optoelectronic functions for PPG wireless NFC monitoring applications, including SpO_2_, HR, and HRV. The device works on the reflectance principle and since it is battery-free, it was possible to achieve a miniature, thin, and flexible construction that can be mounted on a thumbnail or ear lobe. In particular, it uses the reflectance principle in conjunction with near-field communication (NFC) capabilities, which allows operation in a thin, miniaturized, flexible device. A similar flexible device was presented by Polat et al. [90], who designed a patch to record HR, SpO_2_, and respiration. Again, the communication is via NFC, but the photodiode in this case is based on graphene sensitized with semiconducting quantum dots.

Another very important component in the bloodstream indicating health is glucose. The researchers tried to estimate blood glucose levels [91] from green and red light on fingers using machine learning and a random forest regression algorithm. The next approach extracted blood glucose levels by applying four wavelengths of light (green, red, and two IRs) on the wrist using a partial least squares algorithm as the calibration [92]. Both approaches use signal splitting into time frames (29 and 10 s, respectively), and parameterization of time courses also using the Teager–Kaiser energy operator (22 and 24 parameters), and calibration using a reference glucose device. The achieved maximum correlation coefficient of blood glucose determination is 0.91 and 0.86, respectively, which represents a standard prediction error of approximately 6.16 mg/dl.

### 2.3. Calculation of Respiration

The PPG signal is affected by several vital functions and physiological activities, mainly modulated by the heart and respiration. Hence, these activities can be determined by reverse demodulation. Monitoring cardiorespiratory activities using a PPG signal is a well-established non-invasive technique [44,93]. We can use continuous wavelet transform and autoregressive modeling, or a method proposed by Chon et al., which uses variable-frequency complex demodulation and provides slightly better results [94,95]. Breathing manifests itself in PPG in three ways. Pulse wave amplitudes are affected by the blood vessel flexibility, the variation in pulse shape, and a decrease in intrathoracic pressure that leads to increased venous return during inspiration [96,97]. Therefore, we can extract the RR and amplitude of respiration [98,99,100]. PPG technology is used not only in hospitals in intensive care units, during respiratory diseases, or anesthesia, but today it is very often and successfully implemented in wearable health facilities for the general public [101].

### 2.4. Blood Pressure Estimation

Many algorithms are known where systolic and diastolic BP are estimated from the shape of the PPG curve [55,102]. It is possible to use progressive neural learning methods [103,104], FFT-based neural networks [105], or Poincare section analysis [106]. The reliability is continuously growing [107,108,109]. Today, devices that determine blood pressure from the delay of the PPG curve from the ECG signal, the so-called pulse transient time (PTT), have come into use [110,111,112,113]. The correlation of PTT with systolic and diastolic blood pressure is high. By involving multiple wavelengths in the determination, we can even increase accuracy. For example, in a study by Liu et al., where instead of using a single wavelength PPG, three wavelengths were used, the accuracy was increased about two times [114].

Great efforts and promising results are also shown in the extraction of BP without an ECG signal from the so-called local PTT, which is determined by the time difference between the different wavelengths of the PPG signal, for example, from the reflectance mode multi-wavelength PPG sensing at three different locations (fingertip, radial artery and dorsal surface of wrist) using 4 different wavelengths (green 525 nm, orange 595 nm, red 650 nm, and IR 870 nm) [115] or even using 15 different reflectance mode wavelengths and the cross-correlation method [87]. The combination of the described methods, PPG shape recognition and PTT delay methods, processed by deep neural networks, appears to be very promising in the future [77,116]. Today, optical blood pressure monitoring is a complete tool that can be built directly into the firmware or subsequent software, allowing it to be possible to continuously measure blood pressure without using a cuff for virtually any type of PPG sensor. In addition, these algorithms are adapted to analyze PPG signals acquired in different parts of the body and with different sensor topologies [117].

### 2.5. Others

There are also attempts to use PPG signals from wearables for carrier biometric authentication. They assume that the PPG signal is individual for each person and either uses classification algorithms extracting pulse features [118,119], or deep learning frameworks [120,121]. Monitoring absolute cerebral blood flow based on a combination of time-resolved dynamic contrast-enhanced near-infrared spectroscopy and diffuse correlation spectroscopy [122], which uses a configuration similar to multiwavelength (MW) PPG, looks very interesting. In Kooman et al. [123], there are even studies investigating the use of PPG wearables in hemodialysis patients, and Lima et.al. [124] monitored peripheral perfusion. Adhikari et al. [125] developed a multi-wavelength transmission mode PPG method to monitor drug delivery. They examined the concentration of therapeutic gold nanoparticles, quinine, and amphotericin B in mice possessing absorption peaks in the 350–1100 nm range.

A muscle contraction sensor has also been developed to measure signals from intact muscles, which can then be used in robotics and prosthetics [39]. The principle is that the sensor measures the amount of incident light backscattered from the muscles with the intensity of the light varying from whether the muscle is contracted or stretched. The myosin protein in muscle sarcomeres has liquid crystalline properties and is responsible for the anisotropic behavior of muscles [39]. During contraction, the muscle fibers slide along this myosin, and the muscle fiber becomes short and wide, causing anisotropic scattering of incident light. The light that propagates parallel to the muscle fibers is scattered differently to the light traveling perpendicular to the fibers. This anisotropy can be detected utilizing a surface light source and detectors placed longitudinally and perpendicular to the muscle fiber, which represents only a certain modification of the PPG sensor [126,127]. In this research, the authors specifically used a wavelength range from 610 to 700 nm.

**Table 1:** Photoplethysmography sensors.

Sensor type	Application	Sensing Element	Key Parameters	Ref.
Wrist-worn reflectance LED	Motion artifact reduction	PP: Four x OSRAM SFH7050, Motion Sensor: InvenSense MPU9250	Wavelengths 530, 660, 940 nm	[75]
Chest reflectance LED PPG sensor	HR, BP from PPG and PCG	PPG: Osram SFH 7060, PCG ^1^ NXP Semiconductors MPXV7002	Wavelengths 3 × 530, 660, 950 nm	[56]
Body-worn reflectance LED PPG	Skin and muscle perfusion	Eight x LED, Three x PD line/circle configuration	Wavelengths 560, 880 nm	[61]
PPG hands and legs measurement	PPG and ECG system for cardiovascular	Seven PPG probes: STMicroelectronics SiPM detector, Roither LasetTechnick SMC940 LED	Wavelength 940 nm	[63]
Finger-worn organic pulse meter	HR	OLED ^2^, OPD ^3^	Wavelength 625 nm, 46 dB SNR, a constant current of 93.6 µA	[70]
Wrist-worn watch with LED PPG	HR, SpO_2_	PPG: Analog Devices ADPD144RI, Accelerometer: Analog Devices ADXL362	Wavelengths 660, 880 nm, Power consumption 30 μW for a 75 dB, 25 Hz output	[72]
Wrist-worn printed organic	HR, SpO_2_	OLED ^2^, OPD ^3^ multichannel PPG	Wavelengths: Red and IR	[74]
PPG probe	PPG and ECG pattern	OSRAM LT M673 LEDs, STMicroelectronics SiPM detector	Wavelength 529 nm	[77]
PPG reflection sensors	HR, SpO_2_	Maxim Integrated MAX86140/MAX86141	Wavelengths 530, 560, 570, 590 nm	[81]
Ear-worn reflectance LED sensor	PPG during hypothermia	Excelitas technologies CR 50 IRH and CR 50 1M LEDs, 10 BP-BH PD	Wavelengths 658, 870 nm	[128]
Glasses	HR	Reflectance PPG on the nose	-	[84]
Flexible body attachment	HR, BT	Reflectance PPG with thermistor	NFC, placed on wrist, fingertip, temple, or neck	[85]
Flexible body attachment	HR, SpO_2_	Reflectance PPG	Wavelengths 625, 950 nm, NFC, placed on thumbnail, or ear lobe	[12]
Flexible plaster	HR, SpO_2_, RR	Reflectance PPG	Wavelengths 633, 940 nm NFC, graphene PDs	[90]
Mobile phone	HR, RR, SpO_2_	Motorola Droid phone camera	Advanced signal processing	[93]
Laboratory device	HR, ECG, SpO_2_, BP	PPG: HKG-07B, ECG: HKD-10C: Pressure HK-2000 (all Hefei Huake Information Technology)	Integrated PPG, ECG, and pressure pulse wave for cardiovascular disease	[113]
MW ^4^-PPG pad	MW ^4^-PPG spectrometer	15 channels MW ^4^-PPG sensor, plasmonic filters integrated CMOS imager	Wavelengths 505, 510, 515, 520, 525, 620, 625, 630, 635, 640, 930, 935, 940, 945, 950 nm	[87]
Finger-worn reflectance LED	MW ^4^-PPG spectrometer	PPG: plasmonic filters integrated onto a regular photodetector	Wavelengths 515, 630, 940 nm	[87]
Opto-electronic patch sensor	MW ^4^ -PPG movement	PPG: Four channel board Dialog Devices DISCO4	Wavelengths 525, 590, 650, 870 nm	[88,89,115]
MW ^4^-PPG sensor	HR, BP	Four channels MW ^4^-PPG sensor	Wavelengths 470, 570, 590, 940 nm, Sampling frequency 1 kHz	[114]
Flexible wearables	HR, RR, SpO_2_	OLED ^2^, OPD ^3^, POF ^5^ for signal transport	PD current 0.05–4 μA, Signal frequency 0.05–30 Hz	[1,6]
Electrooptical muscle sensor	Muscle contraction	One x LEDs Rodan HIRL 8810, 4x PD Siemens BPW34	Wavelength 880 nm, 4 PDs around LED	[126]
Wearable Organic Optoelectronic	Muscle contraction, SpO_2_	OLED ^2^, PDs	Wavelengths 610, 700 nm	[39]
Sensor for a skin evaluation	Glucose level	C8 MediSensors	Raman spectroscopy sensor	[7]
Finger-worn	Glucose level, HR, SpO_2_	Two x LED, PD, Arduino transmission and reflectance	Wavelengths 525, 615 nm, 22 features from 29 s frames, Random Forest regression algorithm	[91]
Wrist-worn	Glucose level, HR	Four x LED (OSRAM SFH7060 and Vishay VSMY2853), PD (400–1000 nm)	Wavelengths 530, 660, 850, 950 nm, 24 features from 10 s frames, Partial least squares calibration algorithm	[92]

^1^ Phonocardiology; ^2^ Organic LED; ^3^ Organic photodiodes; ^4^ Multi-wavelength; ^5^ Polymer optical fiber.

## 3. Radiation Sensors

In this article, we describe three types of radiation sensors (Table 2). The first two, thermometers and thermal cameras, capture the infrared radiation emitted by the human body. The next one, the Daysimeter, in turn records and measures circadian light exposure, it senses the amount of radiation received by the human body.

Nowadays, radiation sensors are popularly used to detect body temperature in association with infectious diseases [129,130]. These sensors can be found in various forms. The cheapest form is a passive infrared (PIR) sensor used in handheld non-contact thermometers [131]. Another type of radiation sensor is a thermal camera. The only difference between a thermal camera and a PIR sensor is that a thermal camera captures multiple points while a PIR sensor senses only one. The basic principle of sensing these sensors use is to measure the intensity of emitted radiation in a limited infrared spectrum [132,133].

Internal body temperature is different from the body temperature of peripherals such as limbs, and the boundary between them is indeterminate and varies with ambient temperature [134]. Core temperature changes are affected by circadian rhythms. In the early morning, the temperature is lower with the greatest increase in the afternoon, which can be up to a 1 °C difference [135,136,137,138,139,140]. Several factors need to be considered when determining internal body temperature by indirectly measuring peripheral temperature [141]. The surface temperature of the skin depends on the vascularity and anatomical location where the measurement is performed [142]. The best places to determine body temperature, the forehead and chest, were resolved experimentally [143]. However, the determination of core body temperature by indirect measurement can be accomplished using several signal processing methods [144,145]. The most used processing method is an artificial neural network, but a Kalman filter can also be used. These methods process data such as temperature at a particular anatomical location, respiration, physical activity, atmospheric temperature, relative humidity, wind speed, and infrared radiation intensity for proper evaluation. From these data, the internal core body temperature is evaluated with a high accuracy of ±0.03 °C [129,140,146,147,148,149]. 

The measurement of the human body temperature, whether it is core body temperature or peripheral temperature, can be divided into two categories, namely continuous measurement and instantaneous measurement. Instantaneous measurement of body temperature is one of the oldest and most common methods of determining a patient’s condition. A single temperature measurement can tell us whether the patient has a fever or hypothermia [150,151,152,153,154]. From continuous measurements of core body temperature, we can determine the circadian cycle of the patient. The circadian cycle is a regularly recurring cycle of core body temperature change dependent on metabolism and physical activity, and when the cycle changes, we can indicate the onset of the patient’s disease and recommend medical examination [134,135,136,139,140]. Another use of continuous measurement of the patient’s core body temperature is to determine the stage of the ongoing disease [155,156].

### 3.1. Infrared Thermometers

During the COVID-19 pandemic, we became acquainted with non-contact PIR thermometers. At that time, these thermometers became part of our daily routine, and several studies discussed measurement accuracy, proper measurement techniques, or anatomical sites for measuring body temperature [131,132,143,144,148,152]. 

Commercially available PIR thermometers work with wavelengths from 8 μm to 14 μm, thus the sensor must be adapted to the radiation wavelength of human body temperature [130,157]. This spectral range is almost identical to thermal cameras, where it is in the range of 7.5–14 μm [146]. Due to infrared thermometers’ compactness, these devices can be classified as wearable devices [158]. Innovative body temperature monitors based on PIR sensors include the Youbiquo easy check, which is one of the miniature personal body temperature monitors with built-in Wi-Fi and Bluetooth [159]. Other innovative thermometers are various SMART bracelets such as Tempwatch [160], which in addition to conventional PPG measurement also allows continuous monitoring of body temperature via a built-in IR thermometer. For unattended body temperature measurement, another research team produced a wall-mounted automatic BT measurement device based on the ARDUINO platform and PIR sensor [161]. Handheld PIR body thermometers such as ARC Instatemp [162], Withings Thermo (Figure 1d) [15], and others [158] which are enriched with wireless communication, have also become among the innovative meters.

### 3.2. Thermocameras

In addition to non-contact thermometers, thermal imaging cameras have also received admiration. They also began to be mass-used for the rapid detection of people suffering from fever during the COVID-19 pandemic. Recently, several studies describing the measurement methodology with a non-contact thermometer as well as the accuracy of data evaluation from this measurement have been published [142,146,151,153,154]. Instantaneous methods of measuring body temperature also include methods that process temperature scans of the patient’s body surface. The basis of these methods is to detect mainly temperature asymmetries from which a wide range of diseases can be indicated. A breast thermogram is one of the most common additional examinations. From this thermal scan, the doctor may indicate potential breast cancer in case of asymmetric breast temperature [163,164,165,166,167,168]. Other thermographic methods include ocular thermography [169,170,171], diabetic feet detection [172,173,174,175], injury status [176,177,178,179], or even respiratory detection based on a change in temperature around the nose. The accuracy of this method is 97%. Thermography can also be used to localize the site of Frey’s disease [180], to determine the proper function of the thyroid gland [181], the stages and type of cellulitis [182], skin cancer [183], inflammation of the teeth or glands in the oral cavity, or to check the condition of the sympathetic nerve after spinal surgery [184].

One of the most massive innovations is the expansion of an economical solution for thermal cameras connected to conventional smartphones. Basically, cell phones contain a lot of hardware that can be used (screen, processor, memory storage). Therefore, simplified modules with minimized electronics (electronics for thermal imager sensors and communication) have been produced, which are connected via a USB to a mobile phone and thus form one unit capable of thermal imaging. The largest manufacturers of such extensions include FLIR, SEEK, Hti, and others [185,186,187]. Interesting solutions for thermal cameras are also glasses with built-in thermal cameras from Axonim (Figure 1f) [17], or AR02T glasses from Dynacom, which display the thermal image on the glass and thus create a kind of augmented reality [188]. Thermal cameras can also be used for photoplethysmography imaging (PPGI) and infrared thermographic imaging (IRTI), where contactless measurement of a wide range of basic vital parameters is performed. The PPGI method is an extension of the PPG method, so we can use PPG algorithms. From PPGI measurements, the algorithm can determine HR, HRV, RR, respiratory variability (RV), and vasomotion activity. The IRTI method records the temperature distribution of the entire observed object. From the time evolution of temperature changes in the nasal region, it is also possible to determine basic vital parameters such as RR and RV. Both monitoring methods represent unobtrusive data acquisition and the ability to evaluate the spatial association between vital parameters and body area. Hence, these methods allow long-term monitoring or monitoring of effects with special local characteristics [189].

### 3.3. Daysimeters

Nowadays monitoring of external influences on the patient is also required, which helps us to identify potential causes of changes in the vital parameters. These devices usually record the intensity and also type of the light radiation that hits the person. We can evaluate the exposure of the human body to sunlight or artificial lighting, where changes in hormone production, circadian stimuli, or circadian levels are monitored [190]. Devices that make such evidence measurements are called Daysimeters. The word ‘daysimeter’ refers to the ability of 24-h or daily dosimeter of this device [191]. From a wider range, we selected a few that can be worn, such as the wearable light and UV data logger by Rhudy et al. [192], the chrome fibers textile for monitoring IR, UV index, and environment radiation temperature by Wei et al. [193], the portable “Eco-Mini” device for ambient light color balance by Fletcher et al. [194], the personal light exposures devices by Bierman et al. [191], Higgins et al. [195], Jardim et al. [196], a simple UV sensor by L’Oréal in the form of a pendant (Figure 1e) [16], a worn miniaturized microclimate station by Pigliautile et al. [197], a tattoo-based UV exposure sensor [198] which contains colorimetric photosensitive dyes and can be read by smartphone camera [199].

**Table 2:** Radiation sensors.

Sensor Type	Application	Sensing Element	Key Parameters	Ref.
IR thermometer	BT	Thermopile array sensor	Range 35–43.2 °C, Accuracy ±0.2 °C, Resolution 0.1 °C, Wi-Fi, Bluetooth	[15]
IR thermometer	BT	PIR sensor	Range 32–42.8 °C, Accuracy ±0.2 °C, Resolution 0.1 °C	[200]
IR thermometer	BT, screening	PIR sensor	Wi-Fi, Bluetooth	[159]
Smartwatch	BT	PIR sensor	PPG sensor for HR and SpO_2_	[160]
Wall sensor	BT, clinical screening	PIR sensor: GY-906 MLX90614	Range −70–380 °C, Accuracy 0.5 °C	[161]
IR spectrometer	IR radiation spectrum	Precision FT-IR spectrometer Vertex 80v	Spectral range 1.33–16.67 μm	[130]
Thermal camera	Emissivity of the human skin	Thermal camera HY-S280 SATIR (uncooled microbolometer)	Resolution 384 × 288, Spectral range 7–13 μm, Sensitivity 0.08 °C	[133]
Thermal camera	Emissivity of the human skin	Thermal camera FLIR B200 equipped with focal plane array microbolometer	Resolution 200 × 150, Spectral range 7.5–13 μm, Accuracy ±2 °C or ±2%, Sensitivity 0.08 °C	[146]
Thermal camera	Thermal analyses, daily rhythm	Thermal camera Fluke TIR-25 Imager	Resolution 160 × 120, 9 fps, Spectral range 7.5–14 µm, Accuracy ± 2 °C or 2%, Sensitivity 0.1 °C	[135]
Thermal camera	BT distribution from thermal face map	Thermal camera FLIR Systems, T5000 (uncooled microbolometer)	Resolution 464 × 348, 30 fps, Accuracy ±2 °C, Sensitivity 0.03 °C, 17 μm pixel pitch	[153]
Thermal camera	BT distribution from thermal face map	Thermal camera Optotherm42 (uncooled amorphous silicon)	Resolution 640 × 480 pixels, 60 fps, Accuracy ±0.3 °C, Sensitivity 0.04 °C, 17 μm pixel pitch	[153]
Smartphone-based thermal camera	Thermal analyses	Smartphone-based thermal camera FLIR ONE Gen 2	Resolution 160 × 120, 9 fps, Spectral range −20–300 °C, Accuracy ± 3 °C or 5%, Sensitivity 0.1 °C	[184,187]
Smartphone-based thermal camera	Thermal analyses	Smartphone-based thermal camera Hti HT 301	Resolution 384 × 288, 25 fps, Spectral range 8–14 µm, Accuracy ± 3 °C or 3%, Sensitivity 0.5 °C	[185]
Smartphone- based thermal camera	Thermal analyses	Smartphone-based thermal camera Seek CompactPRO XR	Resolution 320 × 240, 9 fps, Spectral range 7.5–14 µm, Sensitivity 0.07 °C	[186]
Glasses with thermal camera	IR thermography	Dynacom AR02T wearable thermal camera	Resolution 80 × 60, 9 fps, Accuracy ±5 °C or 5%	[188]
Glasses with thermal camera	IR thermography	Axonim wearable thermal camera	Spectral range 7–14 µm	[17]
Hybrid optical imaging	PPG and IR thermography, HR, RR	Pulse oximeter, IR camera	Hybrid optical technology for monitoring skin perfusion and temperature behavior	[189]
Wearable light data logger	Effects of light on human	Adafruit UV Light Sensor GUVA-S12SD, Adafruit RGB Sensor with IR filter TCS34725	UV, RGB	[192]
Chromic fibers textile	IR, UV, environment thermal radiation	Dual-responsive Janus chromic fibers, color spectrophotometer CS-820N, FLIR A300	Temperature 15–40 °C, UV intensity 0–250 mW/m^2^	[193]
Daysimeter	Radiation exposure	Hamamatsu S1223-01 silicon PD	Sensitivity 0.13 µA/lux	[191]
Optical sensor	Glucose and dehydration	Laser, camera	Temporal changes of reflected secondary speckles produced in the wrist illuminated by a laser with a change in the magnetic	[46]

## 4. Optical Fiber Sensors

Optical fibers (Table 3) are widely used in wearable sensors, especially in chest belts, smart clothing, textiles, pillows, mattresses, etc. They are used mostly in the form of conventional sensors or based on interferometric principles such as fiber Bragg grating (FBG). Due to the high applicability of optical fibers for long-term medical measurements, easy production, long-term stability, and high sensitivity, they have found themselves in the sight of several research groups. Optical fibers can function as inconspicuous flexible systems monitoring human vital signs such as respiration, cardiac activity, blood pressure and flow, oxygen saturation, shear stress, mobility, gait, temperature, and electrolyte balance. The most flexible variants are also capable of monitoring the joints of the human body [201,202].

Chest belts were one of the first wearable electronics used in health and sports care. Nowadays classic chest electric sensors are gradually being replaced by more modern ones that use optical fibers. Smart textiles and clothing for medical applications are experiencing a huge increase in popularity due to efforts to increase the mobility of patients who need to constantly monitor their physiological parameters. Smart textiles are one of the major innovative types of wearable devices. The development of flexible sensors is important in this industry to make fabrics comfortable for the wearer [203]. Several research groups have focused their efforts and made significant progress in the development of smart textiles and the implementation of fiber optic technology [201,204,205,206,207]. Smart beds, pillows, and mattresses are used especially in areas where for some reason it is not appropriate to use other electrical devices, such as MRI due to high electromagnetic field intensities, or in hospitals in intensive care units, where the optical fiber serves not only as a sensing unit but also for signal transmission and creates a distributed network. Scientific advances are also gradually beginning to permeate the home environment.

### 4.1. Optical Fibers

Silica fiber sensors have the advantage of being electrically insulated, resistant to electromagnetic fields, small in size and easy to connect to networks. Hetero-core optical fiber woven together with wool fibers [208] offers a simple and cost-effective system for monitoring HR and RR. Benneth et al. [209] experimented with 400 µm multimode optical fiber (MOF) without coating and obtained a very good combination of sensitivity and SNR. It was the first time that a MOF sensor was used to evaluate pulse wave velocity-time, which is a good prognostic biomarker of arteriosclerosis and systolic blood pressure estimation. Another approach uses a non-core fiber with optimal length as a sensing head, as well as a filter in a ring cavity to construct a type of fiber laser to sensitively detect RR and HR signals from the shifts of lasing wavelength [210]. Nowadays, marked by COVID-19, sensors placed in face masks have also come to the fore. One example is a breathing sensor attached to a thin plastic film in a bending-sensitive oxygen mask distinguishing different types of breathing conditions (Figure 1i) [20]. Silica fibers are often used in the form of micro/nanofibers (MNFs), thus they are capable of guiding light with greater flexibility [211]. By incorporating MNFs into polydimethylsiloxane (PDMS) thin layers, it is possible to produce skin-like optical sensors with ultrahigh sensitivity, low detection limit, and fast response for pressure sensing [212]. MNFs are also interesting for optical trapping and biomolecular detection [213].

Because silica-fiber-based sensors have limited sensitivity, comfort, and safety, alternative solutions are sought. An excellent alternative is polydimethylsiloxane (PDMS) sensors, also called polymer optical fiber (POF) sensors, which are cheaper, lightweight, more flexible, robust and can measure high strain values without damage. Applications in smart textiles such as HR and RR PDMS sensors with a focus on signal processing and analysis in the frequency domain and the application of various filters in research offers errors below 4 bpm and 2 rpm (respiration per minute) with good accuracy applicable in ballistocardiography in different areas of the human body [214]. In another study, researchers integrated D-shaped cross-section plastic optical fiber into an elastic band [215] to create a respiratory sensing system and focus on reliable recording in different states of motion. Motion artifacts were minimalized by signal processing (wavelet decomposition, FFT, and scalogram methods). In addition, experiments with a novel photonic-based platform—a hybrid plasmonic microfiber knot resonator embedded in a PDMS membrane—were performed and high sensitivity was confirmed when sensing wrist/finger pulse and respiration [216]. The obtained sensitivity was more than one order of magnitude higher, as exhibited by traditional electronic devices or other fiber devices, including Bragg gratings. We can also mention the highly flexible POFs [217] used in the chest belts or POFs fabricated using a 3:2 elastomer/gel combination tested in human biomechanical movement analysis, specifically in the evaluation of skeletal joint angles [218].

Interesting research in a recent period has been completed on the application of POF sensors in smart beds. First, a POF is embedded in mattresses to measure respiration and HR to monitor sleep performance, which was tested on 10 subjects [219]. Its absolute error was less than 1 rpm and 2 bpm. In addition, it can distinguish between four behavioral states related to sleep (on a bed, lying, moving, and leaving bed). This approach can be extended to chairs. Another study is the monitoring of a patient´s vital signs through their bedding using multimode POFs placed under the bed linen [220]. Furthermore, it is a smart cushion based on the SFS (single-mode-few-mode-single-mode) structure [221], where the sensing unit is a sandwich structure consisting of a piece of fiberglass mesh, an SFS structure layer, and a PVC layer and can achieve a maximum error of 1 bpm. Research on the prevention of pressure ulcers in sensitive skin conditions that can detect early changes in skin perfusion, oxygen saturation, HR, blood flow, and pressure on the tissue, and subsequent therapeutic intervention in which POFs are embroidered into a moisture-wicking fabric appears to be very advanced [222]. These devices also withstand disinfection with hospital-type laundry cycles and are of a lower static coefficient of friction than commonly used bedsheets. In another piece of research, a smartphone was integrated with a plastic optical fiber for the diagnosis of respiratory diseases [223]. The flashlight acted as a source, the camera as a photodetector, and the 3D-printed connector performed light coupling. Various matrix structures are sometimes used to increase reliability, such as a 4 × 4 matrix embedded in the mattress [224] which is connected to an Arduino board. The use of fiber optic sensors for in vivo pH measurement is possible using ratiometric sensors based on a hybrid sol-gel pH sensing material deposited on a highly flexible POF tip capable of operating in the physiological pH range with a resolution of 0.0013 pH units. The advantages are excellent sensor-to-sensor reproducibility, long-term stability, and short response time [225].

To increase the sensitivity of optical fiber sensors, they are often pre-bent. The first sensing device is a mat embedded with a macro bending optical fiber sensor [226]. The second and third are microbending fiber optic sensors for perioperative pediatric vital signs monitoring placed under a barrier sheet on the operating table that was tested on 10 subjects [227] and intensity-modulated microbending fiber for non-invasive monitoring of respiration in strong electromagnetic interference environments during MRI [228]. The fourth is the chest belt with a single mode-multimode-single mode (SMS) fiber structure, where six different SMS fiber sensors were tested on six subjects using a short-time Fourier transform and achieved a Pearson Correlation Coefficient with *p* = 0.88 [229]. Another approach uses a stretchable and ultrathin optical sensor with a self-assembled wavy microfiber (Figure 1h) [19]. It can monitor a pulse on the wrist with a high signal-to-noise ratio or, with the combination of an ECG, BP derived from pulse arrival time.

There is also a great deal of effort in the challenge of achieving large stretchability and high sensitivity. One experiment is assembling plasmonic gold nanoparticles (GNPs) into stretchable elastomer-based optical fibers with a core/cladding structure with a step-index configuration. This configuration can obtain strong localized surface plasmon resonance effects and can produce strain sensors that can measure large strains with a low detection limit, fast responsivity, and high reproducibility [230]. They can be used in clothing or attached to the skin surface and detect signals from weak levels (such as a wrist pulse) to large motions (joint bending and hand gestures). This research can be applied, for example, in the analysis of motor disorders such as Parkinson’s disease. Another interesting method is to coat fiber sensors with graphene and attach them to several joints of the human body so that they can track movements during sports. The obtained data indicated ultrahigh sensitivity, wide sensing range, high reproducibility, and fast response [231].

### 4.2. Interferometers

Interferometric sensors represent a large class of extremely sensitive optical fiber sensors. They primarily function by sensing the phase change induced by light, which propagates along with the single-mode optical fiber. Fiber Bragg grating, Mach–Zehnder (MZI), and occasionally Fabry–Perot interferometers are mostly used for wearable devices. The MZI fiber-optic sensor is primarily a transmission interferometer that also involves two closely spaced optical fibers, where localization is achieved by introducing an additional length element into the sensing optical fiber. If the input and the output interfaces are on the same side of the structure, the optical fibers form two closely spaced loops and the sensing area becomes a small difference in length between the two loops. The Fabry–Perot fiber-optic sensor involves a single monomode optical fiber with a sensing area defined by a cavity containing two mirror surfaces that are parallel to each other and perpendicular to the axis of the optical fiber. Changing the optical path length between the mirrors leads to a shift in the frequencies of the cavity modes [232].

From the application in smart clothing, single-mode in-fibers MZI can be introduced to detect HR, respiration, and pulse wave velocity in the shirt [233]. The device uses a new implanted scheme of inserting discontinuities in the fiber to break the total internal reflection and scatter/collect light. Thus, it is not necessary to have an initial calibration or MZI, fabricated by a couple of up-tapers, in a common single-mode fiber used in a chest belt [234]. Other articles are focused on applications in smart beds, specifically in thin mats [235,236] using twin-core fiber-based sensors fabricated by sandwiching single-mode fiber attached under a mattress with a sensitivity of 18 nm/m^−1^ for monitoring of HR and RR. The other research is fiber inserted between two elastic covering layers with a sandwich structure. They applied a three × three coupler-based differentiate and cross-multiplying method [237] to achieve good reproducibility and accuracy with max. errors 2 bpm and 1 rpm in monitoring HR, HR amplitude, RR, and respiration amplitude. The design was tested on 18 subjects, Bland–Altman analysis was applied for detecting body movement, clinical diagnosis of bradycardia, tachycardia, polypnea, and apnea. We were also very interested in the photonic biosensor, developed to acquire multi-bioelectric signals such as ECG, EMG, and EEG [238]. Where the basic sensing technology is based on a Lithium Niobate MZI modulator, which response to the bioelectric signal by modulating the input light intensity. For interconnections only optical fibers are used, and the signal processing is centralized. This solution allows record bioelectric signals from several locations, without compromising the real-time and long-term monitoring.

Applications are also known where Fabry–Perot sensors based on ethyl alpha cyanoacrylate fibers are used to monitor HR [239]. A new technique for the construction of a microscale extrinsic fiber-optic sensor with a confined air cavity and sub-micron geometric resolution—low-coherence interferometry is especially progressive [240]. The confined air cavity is enclosed between a 3 μm thick pressure-sensitive distal diaphragm and a proximal temperature-sensitive plano-convex microlens segment unresponsive to changes in external pressure. It can also simultaneously measure pressure and temperature. A final example is the temperature-sensitive polymer dome at the distal end of a single-mode optical fiber [241] as an intravascular flow sensor, which allowed time-of-flight measurements by upstream thermal labeling of blood and a new optical active fiber sensing technique based on the laser wavelength demodulation, where no core fiber (NCF) with optimal length is selected to function as a sensing head.

### 4.3. Fiber Bragg Gratings Sensors

FBGs have been one of the most studied optical components in the last decade. They are formed by a periodic modulation of the index of refraction of the fiber core along the longitudinal direction [242]. They are used in various applications for temperature, pressure, liquid level, strain, and refractive index sensing. FBG technology is one of the most admired options due to its simple manufacturing, relatively strong signal reflection, and resistance to optical signal intensity modulation, which is possible due to their spectral coding, as well as excellent multiplexing and self-referencing capabilities allowing addressing of multiple sensors with a single optical cable [243]. FBG sensors are small, portable, yarn compatible, and easy to weave into fabrics, and have become the most promising material for sensing elements in creating intelligent clothing [242,244,245].

The use of Bragg grating sensors in smart textiles is very common. For example, we can use a FBG-based smart textile [246], or a respiration belt with an embedded FBG sensor [18] (Figure 1g) for chest motion analysis. To measure body temperature, we can implant FBG sensors into the fabric by combining large and small pipes, which are combined during manufacture. Thus, they reach a temperature sensitivity of 150 pm/°C, which is almost 15 times higher than in bare FBG. They used a mathematical model of heat transfer and a body temperature weighed model and measured temperature using FBGs distributed to five places [247]. The method for 3D skin temperature mapping based on an improved neural network genetic algorithm-back propagation with an absolute error of 0.11 °C is also interesting [248]. Furthermore, the commercial T-shirts equipped with a single fiber optic housing two FBGs for RR and HR waves’ monitoring, where the FBGs were glued on the textile with silicone rubber were reported [249,250,251]. Jia et al. [252] used an FBG sensor for the radial artery pulse waveform measurement and achieved a sensitivity of 8.236 nm/N. 

The logical continuation is a combination of FBGs and PDMS. FBGs encapsulated inside PDMS can work as a multichannel sensor for body temperature, RR, and HR estimation [253], or evaluation of the cardiac functions from carotid HR waveform [254]. In essence, they compared the accuracy of this approach with FBG either glued on the plexiglass pad [255] or manufactured as a stretchable sensor by embedding sinuous-shaped FBG at an off-center position of a stretchable PDMS substrate, which enables measurement of a tensile strain up to 50%. In addition, due to the off-center configuration of the embedded FBG, the designed SFO sensor can also be used to detect bending and torsional deformations with the capability to discriminate directionally. Such sensors could find a wide range of applications in human-machine interfaces, robotics, and health care [256]. Sometimes researchers combined even 12 fiber Bragg grating sensors into one smart textile [257] to perform a detailed thoracic-abdominal motion pattern analysis and achieved good accuracy of the respiratory period (error 0.2 rpm) and tidal volume (error 0.09 L). A very practical design used a FBG sensor embedded in a soft polymer matrix (to optimize adhesion) for control of breathing and HR in the shooting position of archers [258]. They offer a low mean absolute percentage error for RR and HR estimation (≤1.97% and ≤5.74%).

From the application of FBG in smart beds, we can mention a sensor placed under a patient’s body lying supine in an MRI, which was tested on five subjects [259], or the research of various vital parameters such as HR, RR, and pulse oximetry combined with different textile processes such as weaving, knitting, crochet, and stitching with an optical sensor embedded into the textile fabric [260,261]. Another study mentions an alert system for the care of residents in nursing homes [221] where the residents’ HR, RR, temperature, movement, and bed exit are monitored using an optical network with IoT. The results promise HR and RR with a mean error below 1 bpm and can also reveal a sudden onset of high fever and unexpected bed exit during the nights. In the next research, the plate FBG sensor is mounted inside the cushion, which is on the back of the seat and HR monitoring with a maximum error of ±3 bpm and RR with ±1.2 rpm is possible [262].

It is also fascinating to use five FBG sensors simultaneously in different body areas where the breathing pattern was obtained by combining all outputs using a simple algorithm [263]. The approach where FBG is glued to the silicone membrane of a ventilated mask [264] is also interesting, as well as FBG in a face mask for temperature measurement and with a tip coating whose refractive index changes with humidity and thus evaluation of breath to breath changes is available. In vitro, drug level monitoring has also been demonstrated using a long-term grating coated with molecularly imprinted fentanyl-sensitive polymer nanoparticles (detection limit 50 ng/mL) [265]. 

The application of FBGs in wearables is very wide. An alternative study [266] presents measurement results of multiple vital signals at several pulsating points (temple, finger, ankle, and dorsum pedis). They achieved high accuracy at HR, but disappointing results are calculated when estimating blood pressure. Another multiparametric wearable system based on a double-stranded Bragg grating is monitoring neck movements and breathing activity [267]. The sensing elements were placed on the neck, in the frontal and sagittal planes. The system was tested on five subjects and RR was monitored with percentage errors of ≤6.09% and ≤1.90%, during silent tachypnea breathing. In a different study, Martinek et al. [268] detected RR, HR, and body temperature using the LabVIEW evaluation unit and signal processing using frequency-selective filters. Xiang et al. [269] examined the pressure in a pneumatic compression therapy device. The individual phases of the respiratory pattern in the clinical environment were investigated in another study by Pant et al. [270] and pressure and temperature using diffuse optical broadband link technique based on laser Li-Fi technology were described by Murdas et al. [271]. Blood pressure waveform monitoring and the testing of temperature sensor with absolute percentage error (MAPE) (0.003131%, 0.00216581%, 0.000378%), sensitivity (1 Pm/mmHg 0.5 Pm/mmHg, 18.9 Pm/°C) for diastolic blood pressure (DBP), systolic blood pressure and human temperature were performed in an article by Majeed et al. [272]. In a separate study by Massaroni et al. [273], they even analyze the temperature, respiration, and relative humidity of gases in mechanical ventilation to achieve optimal conditions for patients. The probe consists of a needle in which an FBG sensor coated with a hygroscopic material is incorporated. Relative humidity (RH) and so respiration can also be monitored by depositing graphene oxide onto tilted fiber grating [274]. Thus, it offers an ultrafast response within ∼42 ms and a sensitivity of 18.5 pm/%RH and 0.027 dB/%RH in the range of 30∼80% RH by monitoring the wavelength and intensity of the specific cladding mode resonance. The MRI-compatible multi-sensor is advanced and practical [275], which uses graded-index (GRIN) lenses on the tip of the fiber cables and three light sources for imaging pressure, localization, and temperature sensing during assisted surgery. Pressure sensing is performed by applying a released polymer-metal hybrid membrane having a diffraction grating interferometer readout scheme. Temperature sensing is based on the change in absorption and permeability in semiconductors, and localization of the medical device is acquired based on the magneto-optical Kerr phenomenon. The miniature sensor offers a temperature accuracy of ±0.22 °C, a pressure resolution of 1 mmHg, and a localization resolution of 3 mm.

**Table 3:** Optical fiber sensors.

Sensor Type	Application	Sensing Element	Key Parameters	Ref.
Textile	HR, RR	Woven hetero-core silica OF	Error 4 bpm, SD of 1% on a full scale of 2.3 dB (0.2 N)	[208]
Chest belt	HR, RR, BP, PWT	400 µm multimode OF	Laboratory testing, HRV 2.5%,	[209]
Sensing head	HR, RR	No-core fiber laser	Shifts of lasing wavelength	[210]
Respiratory mask	RR	SMS fiber structure	Power variations in OF, fast and reliable response, long lifetime	[20]
Skin-like wearable	Motion, pressure	Glass MNF in PDMS layer	bending radius 30 μm, fast response for pressure sensing	[212]
Textile	HR, RR	POF sensor	High dynamic range and sensitivity, Error 4 bpm and 2 rpm	[214]
Chest belt	RR	D-shaped POF sensor	RR under different movement st	[215]
Elastic band	HR, RR	Hybrid plasmonic microfiber knot resonator embedded in PDMS membrane	Planar strain 1 ‰, Sensitivity 0.83 kPa^−1^, Minimum 30 Pa, Ascending time 20 ms	[216]
Chest belt	RR	POF sensor woven in textile	Error 3 rpm	[217]
Attachment	Join angle detection	POF sensor (3:2 elastomer/gel)	Strain up to 60% (loss of 30 dB)	[218]
Mattress	HR, RR, activity	POF sensor	Error 2 bpm and 1 rpm	[219]
Smart bed	HR, RR	Multimode POF sensor	Comparative between analysis methods (FTT vs Hilbert trans.)	[220]
Cushion	RR	SFS structure Fiberglass mesh/SFS/PVC layer	Error 1 bpm, Enhanced sensitivity	[221]
Bedsheets	Skin perfusion, SpO_2_, HR, pressure on tissue	POF sensors embroidered into moisture-wicking fabric	Low static friction, Withstands disinfection with hospital-type laundry cycles	[222]
Mattress	RR	4 × 4 matrix structures of POFs embedded in the mattress, Arduino	645 nm and silicon PD, Resolution 2.2–4.5%/N.	[224]
pH sensor	pH	Ratiometric sensor based on hybrid sol-gel pH sensing material deposited on a POF tip	Excellent sensor reproducibility, long-term stability, response time 2 min, drift 0.003 pH (22 h)	[225]
Mattress	HR, RR	Macrobend small core OF	High vibration sensitivity, reliability, and good stability	[227]
Pediatric vital signs	HR, RR	Microbend fiber optic sensor under barrier sheet	Mash structure, monitoring of babies	[226]
Chest belt	RR	SMS microbend fiber structure	Six different SMS fiber sensors were tested on six subjects	[229]
Skin-like wearable	HR, BP	Ultrathin optical sensor based on a self-assembled wavy microfiber combined with ECG	Sensitivity ≈ 257 per unit strain, good repetition (SD 1.7% over 100 cycles)	[19]
Human motion	Motion	Plasmonic gold nanoparticles into stretchable elastomer-based OF with a core/cladding structure with step-index configuration	Strains 100%, Detection limit ±0.09%, Responsivity < 12 ms, Reproducibility over 6000 cycles	[230]
Human motion	Motion	Graphene-coated fiber sensors	High sensitivity, Broad sensing range, High reproducibility	[231]
Vital parameters	BP, BT, RR, HR	FBG twin Fabry–Perot interferometer	Mean values within ±5 mmHg, SD ^1^ ±8 mmHg	[206]
T-shirts	HR, RR, BP	Single-mode fibers MZI	Inserting discontinuities in OF to break total internal reflection and scatter/collect light	[233]
Chest belt	RR	Single-mode fibers MZI	-	[234]
Mattress	HR, RR	Twin-core fiber-based sensor Sandwich single-mode fiber	Sensitivity 18 nm/m^−1^	[235,236]
Thin pad	HR, RR, respiration amplitude	MZI inserted between two elastic layers	Errors 2 bpm and 1 rpm, 3 × 3 coupler based differentiate and cross-multiplying method	[237]
Wearable photonic	EMG, ECG, EEG	Lithium Niobate MZI	Wavelength 1530–1565 nm, Gain 1 to 4 mV/μV, Sensitivity 20 μV	[238]
Device	HR	Fabry-Perot interferometer	Strain sensitivity 2.57 pm/μN, Good responses to LF vibrations	[239]
Probe for intravascular sensing	BP, BT	Fiber-optic sensor with a confined air cavity and sub-micron geometric resolution	Resolution 0.11 mmHg (760 to 1060 mmHg) Resolution 0.036 °C (34 to 50 °C)	[240,241]
Smart textile	HR, RR	FBG sensor	Three volunteers, three locations	[246]
Chest belt	RR	FBG sensor	Tested wavelengths 525, 660, 850, 1310, 1550 nm	[18]
Intelligent clothing	BT	FBG sensor	Sensitivity 0.15 nm/°C (33–42 °C), 15x of the bare FBG	[247]
Temperature mapping	Skin temperature 3D mapping	FBG temperature sensors, light source, circulator, fiber coupler	Absolute error 0.11 °C	[248]
T-shirt	HR, RR	Three FBGs glued on the textile with silicone rubber	Highly stretchable and compressible	[249]
Textile	RR	Two FBGs	Sleep apnea, RR during sport	[250]
T-shirt	HR, RR	FBGs	Sensitivity 0.35 nm⋅L^−^^1^, RR accuracy 0.045 s, Error 2.7 bpm	[242]
Radial artery sensor	BP, pulse waveform	FBG sensor, interrogator, light source, circulator	Sensitivity 8.236 nm/N	[252,254]
Multichannel hybrid FO	HR, RR, BT	Two FBGs encapsulated inside PDMS	-	[253]
Skin-like	Muscle motions	FBG-based SFO strain sensor	Mold dimension 2 × 10 × 20 mm	[256]
Textile	RR motion pattern	12 FBGs	RR error 0.2 rpm, RV error 0.09 l	[257]
Smart bed	ECG, HR, BP, PPG, BT, Inspired O_2_	FBG in fabric	Monitoring patient under magnetic resonance imaging	[261]
Smart bed	HR, RR, BT, motion	FBGs in bed	Alert system for residents, Optical network, Error 1 bpm	[193]
Cushion	HR, RR	FBG mounted inside cushion	The error of ±3 bpm and ±1.2 rpm	[262]
Respiratory mask	RR	FBG bonded over a respiratory mask	Remote and continuous RR monitoring	[264]
Multi-parametric	RR, neck movement	Two custom flexible sensors based on FBG technology	-	[267,268]
Nasal flow	RR	FBGs, cantilevers	Nasal airflow into a cantilever	[269]
Wrist-worn device	BT, BP	FBG	Sensitivity 1 pm/mmHg, 0.5 pm/mmHg, 18.9 pm/°C	[272]
Breath humidity monitor	RR relative humidity	Needle, which houses graphene oxide deposited FBG sensor	Response ∼42 m, Sensitivities 18.5 pm/%RH, 0.027 dB/%RH (30∼80%RH)	[274]
Multi-sensor platform	BT, pressure, localization	Lenses at the tip of OF three light sources: 637, 780, 875 nm	BT precision ±0.22 °C, pressure 1 mmHg, localization 3 mm	[275]

## 5. Biochemical Analysis Methods: Colorimetry, Fluorescence, Luminescence

The optical analysis is also represented in biochemistry (Table 4). In the field of wearable electronics the analyses of sweat, saliva, and tears are mainly performed and in addition to recording their amount, the pH, lactate, glucose, Na^+^, Cl^−^, Ca^2+^, Zn^2+^, and other chemical elements are detected [276]. Compared to amperometric devices, colorimetric, fluorescence and luminescence sensors have the advantages of simple structure, low cost, and portable design usually without power operation. The technical design of the sensing part is not a challenge, they often suffice with a simple mobile camera. However, the challenge is the preparation of chemical detergents that indicate the presence of the analytes by changing their optical properties [74,277,278]. For reducing samples and solvents, microfluidics technology has become prominent in wearable intelligent sensor platforms [279,280,281]. All wearable biochemical sensors have some limitations, such as frequent calibration, readout drift due to biofouling, and biweekly sensor replacement [11]. An interesting project for the future is also the research and development of paper modified with nanomaterials, which offers unique physical and chemical properties which then leads to improved quality of paper-based equipment, low cost, biocompatible surface, easy paper disposal (environmental friendliness), flexibility, and low weight. Its use in biosensors looks promising due to the increased separation of analytes, better color contrast, and anchoring of biomolecules [282]. 

Another area of research that is useful for the future is meta-surfaces with the potential for their development for optical sensors and device. Meta-surfaces can be either structured or unstructured with subwavelength-scaled patterns in the horizontal dimensions. They allow several applications such as achieving strong colorimetry and enhancing fluorescence. Plasmonic meta-surfaces can be prepared by color printing using laser post-writing with sub-diffraction-limit resolution. Nanoimprinted meta-surfaces consist of a 20 nm aluminum film buried in a thin-film polymer that is flexible, economical, and recyclable [283]. Radiation from the red color spectrum is most commonly used in medicine and medical applications. The meta-surface represented by Si nanoantenna array with two quasi–bound-states-in-the-continuum (q-BIC) modes can achieve an ideal Schrödinger’s red pixel [284]. Other colors can be achieved by Si meta-surfaces with various geometries and different lattice sizes of different geometric shapes [285,286]. These materials and structures can be used as cost-effective colorimetric chemical detection devices for biochemical analysis methods or can substitute expensive spectrometers [287]. Meta-surfaces as a „structural color” can achieve strong static or dynamic colorimetry. These are tunable by external stimuli, such as mechanical stress, temperature change, electrical voltage, and optical excitation [288]. Various mechanisms are employed to tune the optical system response, essentially a change in scattered size or orientation and a change in the optical properties of the local environment of optical elements, and material phase transitions. This is applicable in sensory medical applications. Meta-surfaces based on the plasmonic mechanism have high optical losses due to absorption in the metal, significantly limiting real-world applications. Via the realization of a novel nanophotonic platform based on dielectric nanostructures to form efficient nanoantennae with ultra-low light-into heat conversion, is demonstrated an approach that overcomes these limitations. Dimer-like silicon-based single nanoantenna produces both high surface enhanced fluorescence and surface enhanced Raman scattering, while generating a negligible temperature increase in their hot spots and surrounding environment at the same time [289,290].

### 5.1. Colorimetry

Colorimetry refers to the color change of analyte-reactive sensing elements, mostly by measuring the absorbance. Crucial factors of optical specificity and sensitivity are the modified substrates or colorimetric chemical reactions. Wearable devices based on this principle often combine various thin flexible layouts, such as a colorimetric layer, microfluidic construct, and optical analysis modules, which are non-irritating and offer robust interfaces with the human body. The choice of material is important for uniform skin contact, for example, cell metabolism may interfere with the measuredthea. The reaction and irritation of the skin leading to irritating dermatitis can also be a problem. The use of hypoallergenic surgical stainless steel and its alloys can help [11]. Colorimetry is suitable for many photo-medical applications and biomarker detections including electrolytes, small molecules, and proteins. For accurate sensing and self-calibration in different lighting conditions, wearable colorimetric devices often have integrated reference graphic markers [279].

Typical colorimetric optical devices are sweat pH meters. The first presented by Curto et al. [291] is based on a wearable chemical barcode micro-fluidic platform incorporating ionic liquids and can be easily incorporated into clothing, head- or wristband. The second is a textile patch which uses a passive pump to gather sweat and moved it through a pre-defined channel for analysis using optical detectors on the waistband by Morris et al. [292]. A very thoughtful wristband by Escobedo et al. [293] with custom-designed μCAD consisting of a sampling area to collect the sweat, a colorimetric sensing area, a passive pump path, and an absorption pad that allows continuous operation for up to 100 min. The readout module consists of a low-power microcontroller unit with a white LED, a digital color sensor, and a Bluetooth communication module. The fourth is a belt placed on the lumbar region made of cotton fabric treated with an organically modified pH-sensitive silicate (ORMOSIL) together with miniaturized and low-power electronics including a wireless interface [294]. The fifth was a thread/fabric-based microfluidic band by Zhao et al. [295] determining pH and glucose. The sixth nonwoven-textile-based platform is fabricated via a photolithography technique which can measure pH and H_2_O_2_ concentration [296]. The seventh is an advanced platform in which optimized chemistries, microfluidic designs, and device layouts enabled accurate assessments of not only total loss of sweat and sweating rate, but also of quantitatively accurate pH values and sweat temperature, and chloride, glucose, and lactate concentrations across physiologically relevant ranges [297]. An interesting step forward is that of Tang et al., who dyed silk fabric with anthocyanin and used it as a pH sensor with great reusability and stability [298] or Promphet et al. with their cotton fabric dyed by a pH-sensitive mixture of methyl orange and bromocresol green, and lactate enzymatic assay [299]. A facile and low-cost strategy is the paper-based wearable system. The one from Xiao et al. [300] can simultaneously monitor sweat, pH, and lactate. The readout is performed by a smartphone-compatible light-shielding box. Another device is skin-interfaced PDMS microfluidic/electronic systems for simultaneous electrochemical, colorimetric, and volumetric analysis of sweat by Bandodkar et al. (Figure 1j) [21] which used photographing the color reactions and NFC communication with smartphones, and can accurately detect the content of glucose, lactate, and pH.

A different wide range of colorimetric wearable devices are contact lenses measuring glucose in tears. Photonic crystals formed by a face-centered cubic arrangement of colloidal particles in a hydrogel are applied for non-invasive glucose sensing [47]. The newer representatives include, for example, where the sensor is built onto commercial contact lenses (Figure 1k) [22]. The sensing part is fabricated on the surface of a glucose-sensitive hydrogel network using a simple stamping method, and the sensor can detect the reflectance of the primary diffractive light. Smartphones can record this light through applications. The next device for glucose determination is presented by Ruan et al. [301]. During the analysis, the diffracted wavelength has a relative linear correlation to glucose concentration as a result of the dielectric periodicity of the polystyrene particles in a crystalline colloidal array. However, in all such measurements, we must take into account that glucose concentration in interstitial fluid is associated with a lag time as compared to blood glucose [11].

From the socio-economic point of view, chronic wounds’ monitoring is also one important part of colorimetry applications. Kassal et al. [302] constructed a wireless smart bandage based on immobilizing cellulosic particles, covalently modified with a pH indicator dye, in a biocompatible hydrogel. The electronics have built-in RFID and are ready for readout by smartphone. Zhu et al. [303] proposed a multifunctional zwitterionic hydrogel for simultaneous monitoring of pH and glucose in diabetic wound treatment. The pH indicator dye and two glucose-sensing enzymes (glucose oxidase and horseradish peroxidase) are encapsulated in the anti-biofouling and biocompatible zwitterionic poly-carboxy betaine hydrogel matrix. To collect the data, a smartphone camera was used. A similar approach was chosen by Wang et al. [304]. They presented a flexible, self-healable, adhesive, and wearable hydrogel patch for on-demand sweat colorimetric detection usable in point-of-care diagnostics and personalized medicine. This wearable hydrogel patch shows excellent reliability and stability for measuring pH (4–9), glucose (0–2 mM), Cl^−^ (0–100 mM), and Ca^2+^ (0–16 mM) in human sweat through integration with smartphones.

Saliva sensors are another area of colorimetry, for example, a biosensor for the determination of urea in saliva working on the principle of immobilization of urease enzyme together with a pH indicator on a strip of filter paper [305]. The readout is obtained by using the smartphone RGB profiling. Vega et al. [306] developed “The Dermal Abyss” for interfacing with the skin by tattooing biosensors. It combines advances in biotechnology with traditional methods in tattoo artistry. In the present work, they replaced traditional inks with colorimetric and fluorescent biosensors that can report on the concentration of sodium, glucose, and pH in the interstitial fluid of the skin. Gao et al. have developed ultrathin photonic devices by combining colorimetric temperature indicators with wireless flexible electronics. The device is based on thermochromic liquid crystals formed from a large pixel array on a thin elastomeric substrate [307]. The read-out is performed by a digital camera, which analyzes color patterns. The original is the approach chosen by Rodin et al. [308], where they incorporated the biosensor into the standard Samsung Gear 2S™ smartwatch. The black glass of the PPG sensor panel contains a compound that changes optical characteristics in presence of various metabolites (such as water, pyruvate, carbonate, lactate, potassium, sodium, ketones) and the result is calculated using a proprietary algorithm from changes in PPG signal. A good overview of soft wearable systems for colorimetric analysis of biofluids was written by Ghaffari et.al [309]. 

### 5.2. Fluorescence

The main disadvantage of colorimetry is that colorimetric tests exist only for a relatively narrow range of biomarkers. Fluorescence analysis of biomarkers can therefore serve as a good extension. The sensing method is based on changing the fluorescence intensity of dyes containing optically active molecules in response to a change in the concentration of different analytes. To react with physiological analytes and to identify and detect molecules are usually required fluorescent derivatives containing suitable chemical reactive groups. Fluorescence analysis provides short response times, minimal instrumentation requirements, and overall low cost. This method is predominantly used in laboratories. At present, there are already several wearable optical devices using the fluorescence method for health assessment or detection of biomarker concentration [279].

Sekine et al. [23] developed a wearable fluorometric sensor in the form of a soft, skin-interfaced microfluidic system to detect Na^+^, Cl^−^, and zinc (Zn^2+^) from eccrine sweat(Figure 1l). The collected sweat is spontaneously directed to serpentine channels connected to the micro reservoirs pre-filled with the specified ions’ probes, which cause changes in the intensity of the fluorescence excitation. After pairing with the smartphone imaging module, we can monitor sweat biomarkers with an accuracy comparable to conventional laboratory equipment. This system allows for quantitative, rapid analysis that can reveal the exact health status. A fluorescence wearable platform for sweat Cl- analysis is presented also by Xu et al. [310]. The device consists of two fluorescent materials on cotton pieces worn on the skin. In another study, Yang et al. [311] introduces a novel bandage-like wearable detector of nucleic acids. The sensor uses flexible microfluidic recombinase polymerase amplification and offers excellent sensitivity and selectivity with a detection limit of 10 copies/μL. This sensor is likely to be of great significance in the field of online pathogen detection for wounds, tumor biomarker diagnosis, and the detection of epidermal cell molecular lesions. March et al. designed a contact lens for glucose monitoring [312]. Contact lenses are made of liquid hydrogel nanospheres containing tetramethyl-rhodamine isothiocyanate concanavalin and fluorescein isothiocyanate dextran. As the glucose concentration increases, the glucose pushes the dextran out of position, and thus the fluorescent intensity begins to increase. The result is read by the designed hand-held photo-fluorometer and can be very useful in the treatment of diabetes. Further research is a fluorescence-based pH sensor with microfluidic mixing and fiber optic detection for a wide range of pH measurements designed by Moradi et al. [313]. They use a single biocompatible and photostable fluorescence indicator named 8-hydroxypyrene-1,3,6-trisulfonic acid trisodium salt (HPTS). For the excitation they used 100 mW 445 nm blue laser and emission at wavelength 520 nm was detected using an inexpensive IF-D91 photodiode. The excitation and emission lights are transmitted by multi-mode optical fiber, so they can simply and inexpensively detect pH in a wide range from 2.5 to 9.0. Interesting and useful is also the research by Ryu et al. [36] where they demonstrated a compact, inexpensive, and practical fluorescence detection system for lab-on-a-chip applications, consisting of 501 nm commercially available InGaN LED, an organic or silicon photodiode, absorptive dye-coated color filters, polarizers, and an injected polystyrene microfluidic chip. The system can detect the cardiac markers myoglobin and CK-MB from the human plasma. Another interesting research is the determination of urinary human serum albumin on a disposable diagnostic microchip with integrated fluorescence detection based on thin-film organic LEDs with an emission wavelength of 540 nm [37]. After illumination of the fluorescence assay, a strong emission is generated at 620 nm, so we can linearly detect concentrations in the range of 10 to 100 mg/L. Ozcan’s group [314] has developed a portable smartphone-based device to measure albumin concentration in urine. The increased risks of diabetes, cardiovascular, and autoimmune diseases are related to the Cr^3+^ disorder, which can be monitored using a portable fluorescence detection system developed by Zhen et al. [315].

### 5.3. Luminescence

Luminescence refers to the process of absorbing external energy inside an object at the ground state, reaching the excited state, and then releasing the energy through light emission to get back to the ground state. According to the kind of energy absorbed, luminescence can be divided into photoluminescence (PL), electroluminescence (EL), chemiluminescence (CL), and sonoluminescence (SL) [316].

Luminescent optical devices operate on the principle of intensity variations between emitted light and received light which is related to concentration changes of different types of used analyte. Devices that use these concentrations of analytes e.g., oxygen, halides, various metals, bio-fluids and crystals can be measured with great results. Dominantly, these devices are designed for very accurate measurement of those substances. Luminescence-based optical devices are particularly attractive due to their good sensitivity and very high selectivity [279]. Protein biomarkers, such as cytokines, play a key role in the diagnosis and treatment of a wide range of diseases. Compared to colorimetry, fluorescence-based protein assays have advantages such as rapid assay time, higher stability, better detection sensitivity, and can examine multiple analytes simultaneously [316]. 

The first presented device (Figure 1c) is an ultra-thin flexible oximeter array with silicon-integrated circuits that can monitor a pulsatile arterial blood signal [14]. The authors use organic printed optoelectronics, which senses reflected light from tissue. OLED and PD arrays are fabricated on indium tin oxide patterned polyethylene naphthalate substrates. Four red OLEDs and four NIR OLEDs emit light. The red LED coupled with the PDs measures the changes of blood hemoglobin absorbance by infrared emission module and receiving module. Additional green LEDs associated with PDs measure blood density in the vessels on changes in transmittance. This skin-sensor system has the potential to transform the oxygenation monitoring of tissues, wounds, skin grafts, and transplanted organs. A similar approach to measuring pulse oxygenation based on the principle of photoluminescence was used also by Yokota et al. in his ultrathin skin-like systems (Figure 1b) [13]. After the light passes through the body, the OPD can detect red and green light emitted by the polymer LEDs and the device can unobtrusively measure the oxygen concentration on the finger. What is more, seven-segment digital displays and color indicators, with a total thickness of only 3 μm, can visualize data precisely on the body. In further research by Lim et al. [314] transcutaneous O_2_ pressure was monitored. The sensor consists of three components: a luminescent sensing film attached onto skin with carbon tape, OLED, and OPD. All components are integrated in a plane in a bandage-like configuration. Another device by Mohr et al. is a cloth shirt for pH and oxygen monitoring. It uses a combination of two luminescent indicator dyes with one fluorescent reference dye for digital cameras’ imaging. Absorbance and luminescence-based dyes have also been combined, e.g., in fluorescence resonance energy transfer-based optical sensors [317]. Roda et al. [318] integrated luminescence assays using 3D printing into a smartphone platform. Specifically, they report an assay for total bile acids and blood cholesterol. Guo et al. [319] presented a stretchable and multifunctional optical sensor (SMOS) with simultaneous readout of BT and strain for wearable physiological monitoring of the human body. The SMOS consists of a stretchable optical sensing fiber made of polymeric nanocomposites containing lanthanide-based conversion nanoparticles. The temperature is read by radiometric intensity measurements of the dual emission upon NIR excitation and stretching is presented in light transmission. Qi et al. show us developed gel-based luminescent conductive material. These two materials have a perfect combination of various properties such as adjustable mechanical strength, biocompatibility, luminescence, and conductivity. The synergistic collection of these properties means that gel-based luminescent conductive materials benefit from these properties in the application and have the advantage of synergistic properties [320].

**Table 4:** Biochemical sensors.

Sensor Type	Application	Sensing Element	Key Parameters	Ref.
Chemical barcode	Sweat pH	Colorimetric micro-fluidic platform incorporating ionic liquids	Adhesive plaster, sweat rate 0.85 ± 0.41 mg min^−1^ cm^−2^	[291,292,293]
Textile fabric	Sweat pH	Colorimetric sensor based on covalently bonded litmus-3-glycidoxypropyltrimethoxysilane coating, PD	Accuracy 0.5 pH	[294]
Thread fabric	pH, glucose	Integrates hydrophilic dot-patterns with a hydrophobic surface via embroidering thread into fabric	5.0–6.0, 25–80 mM, 50–200 μM Color comparison with reference markers	[295]
Skin-interfaced microfluidic device	Rate of sweating, BT, concentrations of electrolytes	Thermochromic liquid crystal	Full capabilities in measuring sweat loss/rate and analyzing multiple sweat biomarkers and temperature	[297]
Paper-based system coupled with a smartphone	Sweat lactate, pH	Paper-based colorimetric sensors, absorbent pad and paper-based sensor are connected with a hydrophilic silk thread	pH—linear detection range (pH 4.0 to 8.0), sensitivity 10.43Lactate range 0 to 25 mM, sensitivity −3.07 mM^−1^	[300]
Skin-interfaced microfluidic	Sweat glucose, lactate, pH	Colorimetric microfluidic platform combined with electronics	NFC communication	[21]
Textile channel	pH, H_2_O_2_	Colorimetric sensor, absorbent microfibrous nonwoven substrate	Range 3–7 pH, 0.1–0.6 μM H_2_O_2_	[296]
Silk fabric	pH	UV-vis spectroscopy, fabric dyed with anthocyanin	Range 4.5–8 pH	[298]
Cotton fabric	Sweat lactate, pH	Colorimetric sensor	Range 1–14 pH, 0–25 mM lactate	[299]
Contact lens	Glucose	Photonic crystals—face-centered cubic arrangement of colloidal particles embedded in hydrogel	Glucose range 0–50 mM, sensitivity 12 nm/mM^–1^, saturation response time 30 min	[22,301]
Contact lens	Glucose in tear fluid	Photonic glucose-sensing material	0–150 nm shift, glucose level 1–100 μmol/L	[5,7,47]
Smart bandage	Wound pH	Colorimetric RFID-based smart bandage	pH indicator dye, biocompatible hydrogel	[302,303]
Smart bandage with wireless connectivity	pH, glucose, Ca^2+^ in wounds	Flexible, self-healable, adhesive and wearable hydrogel patch for on-demand sweat colorimetric detection	Smartphone based, pH (4–9), glucose (0–2 mM), Cl− (0–100 mM) and Ca^2+^ (0–16 mM)	[304]
Saliva biosensor	Urea in saliva	Colorimetric pH indicator	Sensitivity −0.005 pixels sec^−1^/mgdL^−1^ (10–260 mgdL^−1^)	[305]
Epidermal photonic device	BT	Ultrathin device, combining colorimetric temperature indicators with wireless flexible electronics	Multilayer design for accurate colorimetric evaluation of the TLC materials	[307]
Smart watch	HR, SpO_2_, H^+^, Na^+^, K^+^, Cl^−^	Biochemical colorimetric sensor in combination with PPG sensor of smartwatch	PPG panel contains a compound that changes optical characteristics in presence of metabolites	[309]
Skin-interfaced microfluidic	Sweat Na^+^, Cl^−^, Zn^2+^	Array of microchannels and a collection of microreservoirs pre-filled with fluorescent probes	Smartphone read-out, accuracy matches conventional laboratory techniques	[23]
Skin-interfaced	Cl- from sweat	Two fluorescent materials on cotton piece worn on the skin	Lanthanide metal–organic frameworks (MOFs): DUT-101 and Ag+/Eu3+@UiO-67	[310]
Contact lens	Glucose	Liquid hydrogel nanospheres containing tetramethyl rhodamine isothiocyanate concanavalin and fluorescein isothiocyanate dextran	Read-out by hand-held photo-fluorometer	[312]
Fluorescence pH sensor	pH	Fiber optic detection for wide range pH measurements	Linear (5.7–9.0; 4.2–5.7; 3.4–4.2), Polynomial (2.5–3.3)	[313]
Portable fluorescence detection	Trivalent chromium (Cr^3+^)	LED, fiber, spectrometer	Practically monitored using portable fluorescence detection	[315]
Organic oximeter array	2D oxygenation maps	Organic printed in a flexible array configuration, OLED, OPD	Printed organic electronics	[14]
Organic photonic skin	Tensile strain	OLED, OPD	Extreme flexibility, Device total thickness 3 μm	[13]
Cloth shirt for pH and oxygen monitoring	pH, SpO_2_	Absorbance and luminescence-based dyes combined in fluorescence resonance energy transfer based optical sensors	A wide pH range buffer, visual color changes of indicator dyes from green to red by combining indicator and inert dyes	[317]

## 6. Conclusions

In this review, we conducted an extensive review of optical sensors and devices related to human physiology. We have identified four areas: photoplethysmography, radiation sensors, fiber sensors, and biochemical analyzers. Although we have focused only on the selected and most promising types, the review is quite exhaustive. It is clear that the applications of optical sensors in telemedicine are almost endless. 

However, it can still be moved forward. Areas of constant interest are in the use of camera systems to record physiology [321,322,323,324] and all available optical methods for detecting human stress, which we have also addressed in our research, including the detection of cortisol in eccrine sweat [325,326,327], circulatory shock or vasoconstriction evaluation [328], and related changes in peripheral body temperature and PPG signal [128,329,330]. 

It is obvious that optical sensors are widely usable in various types of devices for measuring a wide range of physiological parameters. Due to their many benefits, they are highly preferred in wearable electronics and are the future of telemedicine. However, the interference of ambient light with signal measurements, the short penetration depth into the skin and other bioliquids are some of the drawbacks of optical bio-sensors that limit their widespread use in the biosensing area. The stability of sensors, especially electrochemical ones, can be a problem, where passivation and biological contamination of the electrode can lead to an incorrect response [203]. On the other hand, a general challenge of using optical fiber sensors could be damage of the system while locating it in the field or within its working lifetime which must be controlled and requires further research in this area [331]. It is also possible to move forward in the field of materials, where innovative materials can improve the interaction of the device with the body and thus improve the delivered signal, such as wearable devices with skin-like properties, which are still under development. Nanomaterials may also be a suitable component of flexible sensors and increase scanning performance, but it is still not entirely clear whether they are completely biocompatible and non-toxic to cells [203]. Flexible battery designs and energy management systems would also contribute to a more comfortable fit [11]. Optimizations related to energy sources lead to a small, flexible, stable, comfortable and ideal source ensuring sufficient energy [203]. The discussion is still ongoing about the protection of personal information and data about patients using wearable devices. Protecting medical data is very important so that they are not misused, and therefore the development and implementation of security features in the device are essential for manufacturers and IT infrastructure. The use of optical sensors can help reduce healthcare costs through the continuous monitoring of physiologically important parameters. For example, athletes have improved their performance [332]. It will be important to improve remote biomedical imaging in the future. Today, we have technologies such as machine learning, artificial intelligence, 5G networks, the Internet of Things (IoT) that help improve the technical side, and we will certainly be implementing it in future research [333]. Ultimately, all research leads to more stable, accurate, robust sensing mechanisms and components that will withstand long-term use [203]. We believe that this study opens some possible springboards for further research.
